# Advancement of Scaffold-Based 3D Cellular Models in Cancer Tissue Engineering: An Update

**DOI:** 10.3389/fonc.2021.733652

**Published:** 2021-10-25

**Authors:** Kavitha Unnikrishnan, Lynda Velutheril Thomas, Ram Mohan Ram Kumar

**Affiliations:** ^1^ Department of Cancer Research, Rajiv Gandhi Center for Biotechnology, Thiruvananthapuram, India; ^2^ Division of Tissue Engineering & Regenerative Technology, Sree Chitra Thirunal Institute of Medical Sciences and Technology, Thiruvananthapuram, India

**Keywords:** 3D scaffold, cancer, cellular interaction, tissue engineering, extracellular matrix (ECM)

## Abstract

The lack of traditional cancer treatments has resulted in an increased need for new clinical techniques. Standard two-dimensional (2D) models used to validate drug efficacy and screening have a low *in vitro*-*in vivo* translation potential. Recreating the *in vivo* tumor microenvironment at the three-dimensional (3D) level is essential to resolve these limitations in the 2D culture and improve therapy results. The physical and mechanical environments of 3D culture allow cancer cells to expand in a heterogeneous manner, adopt different phenotypes, gene and protein profiles, and develop metastatic potential and drug resistance similar to human tumors. The current application of 3D scaffold culture systems based on synthetic polymers or selected extracellular matrix components promotes signalling, survival, and cancer cell proliferation. This review will focus on the recent advancement of numerous 3D-based scaffold models for cancer tissue engineering, which will increase the predictive ability of preclinical studies and significantly improve clinical translation.

## Introduction

**Figure 1:**
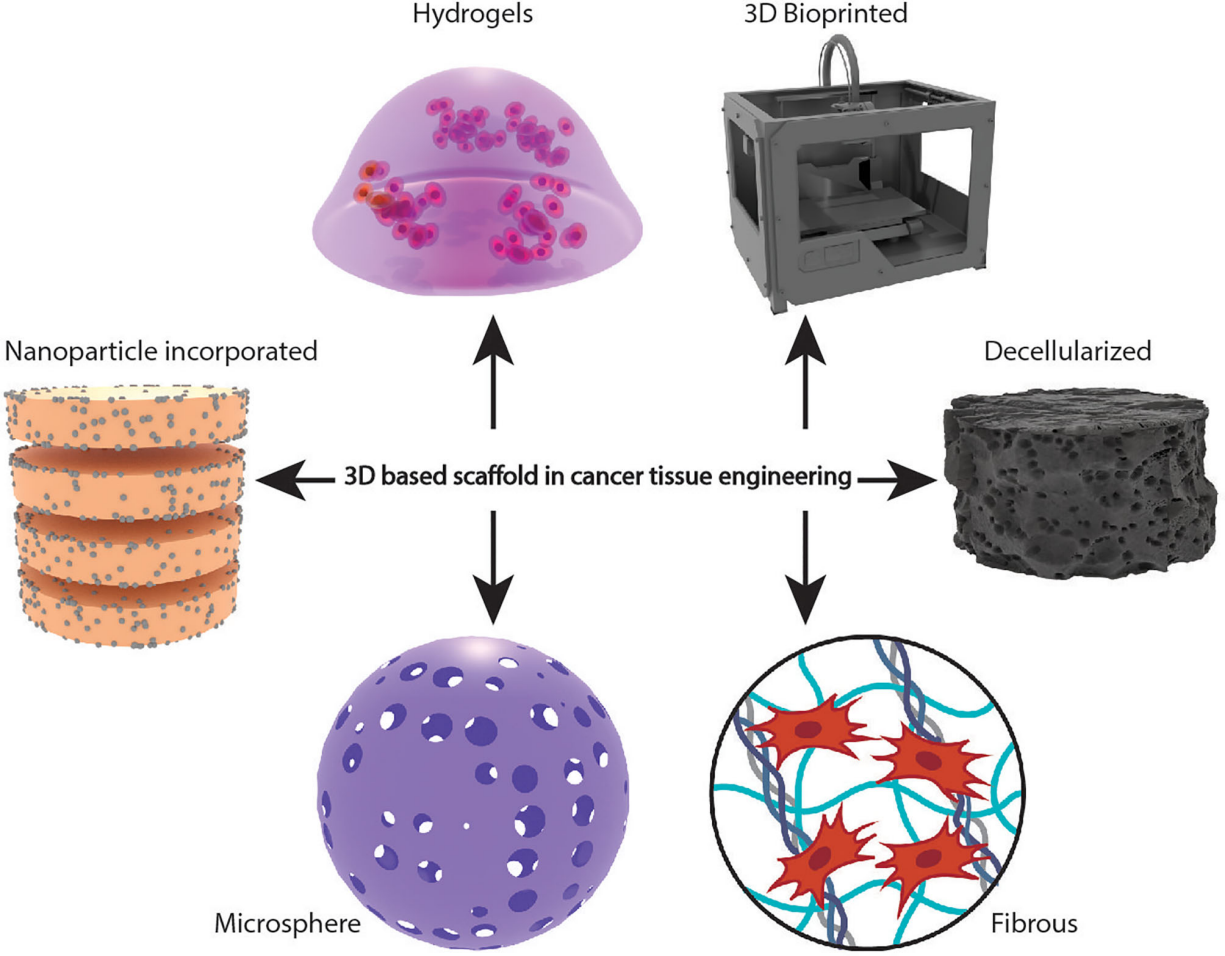
Schematic representation of different types of scaffold-based 3D cellular models in cancer tissue engineering.

Two-dimensional (2D) cell cultures have been traditionally applied in cancer research and are still a dominant culture method in many biological studies. Cell-based assays are essential in the drug discovery and validation process, and 2D cell culture offers a platform for investigating cell and tissue physiology and disease outside of the organism (1). Due to the significant disparities in the cellular environment, 2D cell cultures cannot perfectly replicate or reproduce the *in vivo* conditions. Since 2D cultures have unnatural growth kinetics and cell attachments, natural microenvironments of the cells are not fully represented (2). Compared to *in vivo* environments, cells on 2D culture plates exhibit altered proliferation, behaviour, and reaction to toxicants (3). Hence, there is an absolute necessity to develop conditions that mimic human physiology. The most common type of three-dimensional (3D) tissue culture employed are cell spheroids. Spheroids are cell clusters used for imitating the tumor environment and angiogenesis. However, the inadequacy of their development, the lack of tissue extracellular matrix (ECM) components, and their questionable biological significance make them not considered appropriate cancer models (4). The 3D-based scaffolds can influence the mechanical and biochemical signals critical for facilitating cell-cell and cell-ECM interactions and mimic the hypoxic and nutrient deprivation conditions of the native tumor microenvironment (TME) (5, 6). The use of 3D based scaffold models for studying the complex interactions between the cells and TME has gained sufficient attention. 3D based scaffolds are excellent models for culturing primary patient-derived cancer cells, screening different drugs, testing drug efficacy on patient samples, and thereby paving the way for personalized therapies (7–9). These models are also used for co-culturing normal and malignant cells, recapitulating the tumor heterogeneity and are utilized to elucidate the role of stromal cells on the hallmarks of cancer (9–12). Biomaterials in various forms, such as hydrogels, solid scaffolds, decellularized original tissue, etc., increase culture efficiency and cell functions, making them ideal 3D based scaffold models (**Figure 1**).

Many 3D scaffold culture systems are available, which have been created using a variety of polymers, and the activities of tumour cells in scaffolds have been studied (**Table 1**). The knowledge of 3D culture methods has grown substantially, resulting in various applications in cancer research. In this review, we will discuss the different types of 3D scaffold-based systems and their usefulness in cancer research and clinical settings.

**Table 1:** Advantages and disadvantages of different scaffolds in tissue engineering.

Type of scaffolds	Advantages	Disadvantages	References
Hydrogels	• Tissue-like responsiveness • Water-soluble factors are easily supplied to cells • Generally biocompatible • Low immunogenicity	• Mechanical resistance is minimal • Physically cross-linked gels are weak	(13, 14)
3D Bioprinted scaffolds	• High-reproducibility of biomimetic microenvironments • Homogeneous distribution of cells	• Low-concentration solutions can be a limiting factor when building up material into a 3D structure.	(15, 16)
Decellularized scaffolds	• Provides ECM environment • High bioactivity, • Low immunogenicity • Promotes cell-material interactions	• Decellularization of thick tissues can be difficult. • The number of cell adhesion sites is limited.	(17, 18)
Fibrous scaffolds	• Characterized by high surface-area-to-volume favouring cell proliferation, migration, adhesion and differentiation of cells	• Low structural stability • Limited by cell seeding • Scaffold morphology is difficult to regulate. • Limited in thickness and small pore size.	(19, 20)
Microsphere scaffolds	• Cumulative release of encapsulated bioactive substances • Long-time maintenance of cancer cells in culture • Excellent mechanical properties	• May results in loss of bioactivity of encapsulated factors • Residual solvent toxicity • Expensive	(21)
Nanoparticle incorporated scaffolds	• High penetration ability • Tunable surface properties	• Particle aggregation	(22, 23)

## Hydrogel-Based 3D Scaffolds as *In Vitro* Cancer Models

Hydrogels are three-dimensional networks made up of hydrophilic polymers that bind through covalent bonds. They are polymers that are capable of capturing vast quantities of water and retain a 3D structure. These scaffolds have excellent biocompatible, biochemical and biophysical tunability. Drug response and cell function are influenced by the cellular structure within the hydrogel networks (24). Due to these hydrogels’ highly porous and hydrated nature, encapsulation of cancer cells is possible, which can be easily analyzed and can assess cell viability, proliferation, tumor formation, or onset of hypoxia. Due to its capacity to encapsulate cells and imitate native ECM, hydrogel-based 3D scaffolds gain popularity in cancer research. Natural based polymer systems have found much potential as hydrogel scaffolds for cancer tissue development. Gelatin Methacrylate (GelMA) and Matrigel hydrogels are such systems that promote 3D cellular proliferation and enable nutrients to diffuse through their network (25). Both of these hydrogels act as ECM-mimetic platforms in various disease models. Polymers have innate cell responsive sequences within their molecular structures, providing a niche for cancer cell attachment and growth. Recently, Monteiro and co-workers evaluated the *in vitro* maturation of MG-63 osteosarcoma spheroids and cell lines in GelMA and Matrigel hydrogels. MG-63 spheroids in both hydrogels exhibited significantly higher invasion and high sensitivity to Lorlatinib when compared with the cell-laden counterparts. After embedding cells and on day 14, spheroids invaded the surrounding Matrigel and GelMA matrix, recapitulating late-stage tumor features. Moreover, the use of cell-laden hydrogel models may present limitations while evaluating drug resistance and anti-metastatic functions (26). Ovarian cancer cell line, HO-8910PM, exhibited significant cell proliferation and active cell growth in RADA16-I peptide hydrogels composed of natural amino acids. Cells cultured on RADA16 hydrogels had elevated integrin levels of integrin β1, E-cadherin and N-cadherin. They showed significantly higher chemoresistance to cisplatin and paclitaxel with 2D culture, making it an excellent 3D ovarian cancer *in vitro* model (27). Collagen-based hydrogels are presently explored to understand the cancer cell viability and invasive properties. Liu et al. showed that ovarian cancer cell lines, OV-NC or OV-206 cultured in collagen I hydrogel scaffolds gradually turned to multicellular spheroids with increased cell viability and enhanced expression of epithelial to mesenchymal transition (EMT) markers, vimentin, fibronectin, and N-cadherin. They also expressed a significant upregulation of the signalling pathways, Wnt/β-catenin and TGFβ/Smad, to induce EMT (28). However, these natural-based scaffold systems have a higher biodegradation rate due to MMP responsive cleavage moieties that disrupt tissue formation. Synthetic polymer-based hydrogel systems like Polyethylene glycol (PEG), Polyvinyl alcohol (PVA), Polyvinyl pyrrolidone (PVP), etc., although inert, have been advantageous over natural systems, providing better mechanical strength and stability. The development of synthetic hydrogels like PEG, PVA or polymers of vinyl monomers promotes mechanical and chemical properties of cancer cells. Pradhan et al. developed PEG-fibrinogen hydrogel models as a potential ECM scaffold for breast cancer. Breast cancer cells, MCF-7, SK-BR-3, MDA-MB-231, exhibited high viability and stiffness-dependent variation in morphology and colony size in the PEG-fibrinogen hydrogel model (29). Livingston et al. used PEG hydrogels to encapsulate estrogen-driven breast cancer cells, MCF-7, and compared the cell viability and cell adherence with naturally derived hydrogels such as Matrigel. No significant changes regarding cell viability and cell adherence were observed; however, proliferation was significantly higher in PEG hydrogels (30). Microfluidics system is an excellent tool in recapitulating the behaviour of cells and tissues *in vitro*. Anguiano et al. developed microfluidics system filled with hydrogels of mixed collagen-Matrigel composition to determine the migration of lung cancer cells under different cancer invasion microenvironments. In their study, migration phenotype and dynamics of lung cancer cells increased in microfluidics platform filled with hydrogels. The use of these devices will allow the study of invasion strategies in different environments as well as define efficient therapeutic anti-cancer drugs (31). Lee et al. integrated hydrogels within a microfluidic chamber for studying tumor cell attachment and migration properties. The circulating tumour cells were isolated from prostate cancer patients and introduced to the hydrogel microfluidic chamber. The cell laden hydrogels from the chamber was then directly implanted into mice with preserved xenograft capacity which enhanced tumor cell growth (32). Synthetic hydrogel systems do not have any cell responsive sequences that support cell growth; hence, most of these systems are modified to make them more cell responsive, like conjugation of cell-responsive peptides such as RGDS which promote adhesion between substrates and cells, IKVAV which helps in facilitating cell adhesion, tumor growth, migration, and angiogenesis (33) and YIGSR which promotes cellular adhesion (34). A balance must be maintained while working with hydrogel systems to support the gel’s mechanical integrity and enable viable cell encapsulation efficiency.

## 3D Bioprinted Scaffolds as *In Vitro* Cancer Models

3D printing is a computer-aided designed structure that generates viable 3D constructs. It has emerged as an *in vitro* tumor- mimicking model to investigate the biological mechanism of tumor development. 3D bioprinting is evolving rapidly than other 3D scaffold production techniques for replicating the architecture and microenvironment of tumor tissues. Extrusion-based bioprinting (EBB), droplet-based bioprinting (DBB), and laser-based bioprinting (LBB) are the three bioprinting modalities (LBB) that are commonly used. EBB is based on the robotic dispensing of a continuous stream of bioinks powered by pneumatic or motor forces. The DBB modality is based on droplet deposition under mechanical actuation, thermal, piezoelectric, or solenoid-based. LBB constructions are created by depositing bioinks in a pattern determined by a laser path. A 3D construct with a bioink comprising cells encased in a hydrogel is printed in a scaffold-based manner (35). Recently, Wang et al. elucidated the mechanism of angiogenesis and the origin of tumor vascularization of glioblastoma (GB) in a 3D bioprinted hydrogel scaffold. GSC23 GB cells exhibited a more remarkable ability to form cell spheroids, secretion of VEGF-A, and formed tubule-like structures in the bioprinted hydrogel scaffold (36). Kim et al. developed a bladder cancer 3D scaffold model using GelMA hydrogel constructed with a 3D bioprinter. Bladder cancer cell lines, 5637 or T24 cultured in the 3D bioprinted scaffold showed an increased cell proliferation and cell-cell interaction compared with the 2D culture. The anti-cancer drugs rapamycin and Bacillus Calmette-Guerin (BCG) reduced cell proliferation rates in 2D conditions but could not reproduce the same results in the 3D models. The possible reason for this discrepancy is that the amount of cytokines released in response to drug treatment is higher in 2D conditions but minimal in 3D systems. The efficacy of both drugs on the scaffold model showed an increased drug resistance and less sensitivity than in the 2D culture (37). One of the limitations in 3D bioprinting is the availability of the suitable bioink, bioprinting time and dimension of bioprinted tissues to produce cancer-mimetic models appropriate for industrial use. The choice of bioink is essential in recreating an *in vivo*-like tumor environment since the TME is a complex matrix whose content and dynamics not only evolve geographically but also change with the kind of tumor and stage of the disease. Choosing a suitable bioink is one of the most crucial prerequisites for mimicking the tumor microenvironment. High resolution during printing, *in situ* gelation, visco-elastic properties, low cost, readily available, industrial scalability, biomimicking tissue internal structures, mechanical integrity, short post-printing time for maturation, and immunological compatibility when implanted *in vivo* are some of the other essential desirable factors for a bioink (38). Oxygen gas permeability, metabolic waste permeability, and nutrition transport are also significant while considering the bioink (39). **Table 2** lists the established 3D bioprinted scaffolds for each cancer type.

**Table 2:** Bioprinting substrates and techniques employed for each cancer type.

Sl. No	Cancer type	Cell lines	Substrate	Bioprinting techniques	References
1	Breast	MCF-12A, MCF10A	Rat tail collagen I	EBB	(40)
		MCF-7	Magnetic bioink	Diamagnetophoresis	(41)
		MCF10A, MCF10A-NeuN, MDA-MB-231, and MCF-7	Matrigel and gelatin-alginate	EBB	(42)
		MDA-MB-231	Polyethylene glycol	LBB	(43)
		MDA-MB-231	Hydroxyapatite nanocrystals	EBB	(44)
		MDA-MB-231	Poly(lactic acid)	FFF	(45)
		MCF-7	Gelatin	EBB	(46)
2	Ovarian	OVCAR-5	Matrigel	DBB	(47)
		SKOV3	GelMA	LBB	(48)
3	Cervical	HeLa	Gelatin	EBB	(49)
4	Neuroblastoma	SK-N-BE (2)	GelMA	–	(50)
		SK-N-BE(2)	Sodium alginate hydrogel	FRESH	(51)
		SH-SY5Y	Calcium-induced crosslinking of alginate	EBB	(52)
		SH-SY5Y, HUVEC, Mesenchymal stromal cells	Collagen type 1 and agarose	DBB	(10)
5	Colorectal	HCT116	Collagen 1	EBB	(53)
6	Osteosarcoma	SaOs-2	Ti3C_2_	–	(54)
7	Glioma	U118	Gelatin and sodium alginate	–	(55)
8	Lung	A549, 95-D	Gelatin and sodium alginate	EBB	(56)

FRESH, freeform reversible embedding of suspended hydrogels; FFF, Fused Filament Fabrication.

## Decellularized Scaffolds as *In Vitro* Cancer Models

Decellularized scaffolds are made by decellularizing organs to remove cellular components and form an acellular ECM. Decellularized tissue-based 3D scaffold models have an advantage over other 3D models. They mimic the tissue-specific ECM composition, providing the biochemical cues needed for cell-ECM interactions and help identify the patient-specific response to anti-cancer drugs. Many models have recently been developed to study tumor progression, validate anti-cancer drugs, and assess drug resistance. Leiva et al. used a patient-derived scaffold (PDS) generated from breast cancer samples to understand the changes in the TME of MCF7 cells in response to chemotherapy. Increased drug resistance was observed in response to chemotherapeutic drugs such as doxorubicin and 5-fluorouracil. Upon varying drugs and drug concentrations, the model showed differential expression of markers associated with cell proliferation (MKI67, CCNA2), EMT (VIM, SNAI1), and cancer stem cell (CSC) phenotype (NANOG, POU5F1, CD44, ABCG2) (57).

Similarly, colorectal cancer cell lines, HT29, were grown in PDS generated from colorectal cancer samples. The transcriptomics and proteomics profiles of the PDS cultured cells were similar to those of patients with colorectal cancer. The model was capable of recapitulating the individual patient TME, and the study proved that the TME of individual patients has a role in tumor progression, making them an excellent model for personalized preclinical testing (58). Li et al. developed a lung scaffold model for studying breast cancer cell proliferation and drug resistance. MCF7 cells cultured in a decellularized porcine lung scaffold showed an increased cell proliferation and increased drug resistance in response to 5-fluorouracil. Certain features of lung scaffold, porous alveoli-bronchiole, collagen fibres, and the hypoxic condition were similar to the *in vivo* TME. The porous alveoli-bronchiole provided a large surface area for initial cell attachment, and the native collagen fibres increased cell-cell and cell-ECM interactions (6). To identify the impact of each protein component of ECM in tumor progression, Wishart et al. used a decellularized ECM scaffold from obese and tumor bearing mice mammary glands. They showed that the decellularized ECM obtained from obese and tumor- bearing mice mammary glands promotes triple-negative breast cancer (TNBC) invasion by upregulating collagen VI and EGFR/MAPK signalling (59). Natural tissue scaffolds can recapitulate the patient-specific conditions and the cell-ECM interactions. Still, the scaffolds are easily degradable, and it is difficult to alter the physical and mechanical properties of the scaffold.

## Fibrous Scaffolds as *In Vitro* Cancer Models

The *in vivo* tumor microenvironment is naturally highly heterogeneous. The native ECM plays a significant role in maintaining tumor heterogeneity. Many fibrous scaffold models were developed to recapitulate the native ECM fibrous architecture. Electrospinning is one such process of fabrication for generating nanofibrous scaffolds that are highly porous. It involves using high voltage to create charges on a polymer solution that is ejected at a particular flow rate using a syringe pump. Once the electrostatic forces generated exceed the fluid’s surface tension, it is pulled towards a grounded collector and fibres are formed in the process. Volatile solvents are used to remove the solvent as the polymer fibres are pulled towards the collector system (60). A recent study by Permlid et al. developed a highly porous 3D scaffold using 3D electrospun Poly (ϵ-caprolactone) (PCL) fibres capable of mimicking the collagen fibres present in the ECM. Two non-malignant cell lines (Human adult dermal fibroblasts and MCF-10A) and two malignant breast cancer cell lines (MCF7 and JIMT-1) were co-seeded on the fibrous mesh. All the cell lines showed penetration into the fibres. The non-malignant cell lines spread between fibres and formed elongated structures, whereas the malignant cell lines aggregated together and formed spheroids. This scaffold could be used to analyze the impact of stromal cells on cancer progression (11). Dondajewska et al. performed co-culturing of the breast cancer cell line, EMT6, and fibroblast cell line, NIH3T3, on a silk fibroin scaffold. They observed a significant increase in ECM production and upregulation of markers associated with EMT and cancer-associated fibroblasts (CAF) (12).

Poly (lactic acid) (PLA) polymers are also employed for developing fibrous scaffolds. Polonio-Alcala et al. cultured TNBC cell line MDA-MB231 on an electrospun PLA fibrous scaffold. The cells exhibited a higher rate of proliferation than in the 2D conditions. However, no significant difference was observed in the levels of EMT related genes, vimentin, snail, and E-cadherin, indicating that the cells maintained an epithelial phenotype rather than acquiring a mesenchymal phenotype. Another limiting factor is that the cells had a reduced CSC enrichment in electrospun PLA fibrous scaffolds. The CSC marker SOX2 showed an increased expression over three days of culture but downregulated at six days of culture (61). The breast cancer cell line, HCC1954, cultured on electrospun PCL, was characterized by an increased CSC population and mucopolysaccharide production. Compared to the 2D culture, a decrease in drug sensitivity to doxorubicin and electroporation/bleomycin was also observed in the 3D scaffold model. The possible reason for a reduction in sensitivity might be the abundant CSC population or ECM presence (62). The synthetic polymers, PCL and PLA, were capable of mimicking the structural properties of native ECM, but they failed in providing the biochemical signals needed for cell-ECM communication.

The hybrid fibrous scaffold models are employed in many cancer types. Pal et al. developed a 3D hybrid scaffold model composed of Poly Lactic-co-Glycolic Acid (PLGA) fibres and GelMA hydrogel, which recapitulates the *in vivo* ECM better than GelMA or PLGA scaffold alone. The gastric cancer cells, MKN74 or the breast cancer cells, MDA-MB231 cultured in the hybrid scaffold showed a heterogeneous behaviour in which a portion of cells proliferated, another small part underwent EMT, and a few cells showed cancer stem cells like phenotype. The heterogeneity of this model makes it worthwhile to study cancer cell proliferation, EMT, and enhancement of cancer stem cells in breast and gastric cancers (63). Murakami et al. developed a 3D scaffold system using silica fibre of unwoven sheets called cellbed. Squamous carcinoma cells cultured in Collagen IV coated cellbed scaffold showed an increased cell proliferation and invasion (64). The hybrid fibrous scaffold models are a more realistic model that mimics the cell-ECM interaction better than the synthetic fibrous scaffold alone. They can validate anti-cancer drugs and elucidate the role of particular ECM proteins in tumor progression. Fibronectin is one of the most abundant ECM proteins and is found overexpressed in many cancers. Jordahl et al. developed a 3D fibronectin network scaffold using fibrillar fibronectin and PLGA microfibers. They observed that the engineered fibronectin network promotes breast cancer cell proliferation, invasion, EMT and *in vitro* expansion of primary patient-derived breast cancer cells (7). In addition to solid tumors, hybrid fibrous scaffolds have been employed to study hematologic malignancies. Acute lymphoblastic leukaemia (ALL) Jurkat cells cultured in collagen type 1 coated PCL scaffold showed increased cell proliferation and drug resistance to daunorubicin and cytarabine than the 2D culture and PCL alone scaffold. The phenotype of leukemic cells remained unaltered but there was an upregulation in the level of the transcription factor STAT3 and discoidin domain receptor 1 (DDR1) (8). Nair et al. developed a polyurethane (PU)/poly-L-lactic acid (PLLA) micro-nanofibrous scaffold by a thermally induced phase separation technique. Acute Myeloid Leukemia (AML) cells cultured in the scaffold showed an increased cell adhesion and drug resistance (65). Phan Lai et al. used hybrid 3D chitosan-alginate fibre scaffolds for the *in vitro* evaluation of tumour−stromal−T cell interactions. By employing this 3D scaffold model, they showed that cancer-associated fibroblast (CAFs) modulated the ability of specific T lymphocytes to kill breast cancer cells *via* TGF-β and IL-10 pathways. The scaffold helped to determine the function of CAFs in modulating the immune response in a model of breast cancer (66).

Fibrous polymer scaffold-based dual-functional scaffolds are gaining clinical importance. Hou et al. developed a dual-functional scaffold using PCL fibres and graphene for bone cancer treatment and regeneration. The scaffold was designed to make the outer layer made of PCL and graphene and internal PCL layers. They observed that graphene could provide mechanical support to the scaffold and inhibit cancer cell proliferation. The PCL layers were capable of bone regeneration by recruiting healthy cells and enhancing cell attachment, proliferation, and differentiation (67). Overall, the PCL/graphene dual-functional scaffolds are a novel clinical approach for bone cancer treatment and regeneration

## Microsphere/Microparticle Scaffolds as *In Vitro* Cancer Models

Scaffold pore size and pore interconnectivity determine the amount of oxygen and nutrients distributed over the scaffold. Microspheres/microparticle scaffold fabrication provides uniform pore size and pore interconnectivity, and increased surface area. Microsphere fabrication technique has been mainly used in drug delivery systems to achieve the maximum and controlled delivery of drug moieties. In scaffold development, they have been used to build blocks of a more extensive 3D scaffold system. Most of the scaffolds developed in the area of cancer model use the injectable soft microsphere scaffolds instead of the sintered ones. Kuriakose et al. developed three PLGA microparticle based scaffolds for growing lung cancer cell lines, A549. The PLGA microparticles were prepared using the porogen-gelatin, sodium bicarbonate (SBC), and poly N-isopropylacrylamide particles. Upon comparing the three models, all three were stable and biodegradable. But the PLGA-SBC based model with a relatively larger pore size and better pore interconnectivity favoured cell attachment and proliferation (68). Optimal pore size and pore interconnectivity facilitate uniform distribution of oxygen and nutrients that positively affect uniform cell attachment. Damecha et al. developed a porous PLGA microsphere (PPMS) based scaffold with large, uniform, and interconnected pores using alginate microsphere (AMS) porogen. Later, PPMS was coated with collagen and co-cultured with lung cancer cell lines, A549 and MRC5. The cells in the scaffold showed an increased cell attachment, proliferation and drug resistance in response to the anti-cancer drugs, doxorubicin, cisplatin, curcumin, paclitaxel, etoposide, and gemcitabine when compared with the 2D culture. The collagen-PPMS scaffold model is a potential *in vitro* lung tumor model which could be used for evaluating cancer progression, screening drugs, and elucidating the mechanism of drug resistance (69).

## Nanoparticle Incorporated Scaffolds as *In Vitro* Cancer Models

The nanoscale dimension characterizes the nanoparticles. The unique size of nanoparticles gives it a high surface to volume ratio facilitating the efficient transport of oxygen and nutrients on the scaffold and finds potential application as a delivery system in scaffolds. Nanoparticles are generally combined with the existing 3D scaffolds. The nanoscale dimension of the particles enables the 3D scaffolds to improve their physical and mechanical properties. Tornin et al. developed a porous 3D bone-like scaffold using hydroxyapatite nanoparticles (nHA) and collagen1 (Col1) that mimics the osteosarcoma microenvironment. Human osteosarcoma cells, MG-63 cultured in Col1/nHA scaffold showed an increased expression of fibronectin, MMP2, and MMP9. The scaffold also favoured osteo mimicry of MG-63 cells by enhancing the expressions of genes, osteocalcin, BMP-2, RUNX2, and alkaline phosphatase involved in bone cancer malignancy. Previous studies have shown cold plasma-activated ringer’s solution (PAR) as a potential therapeutic approach against osteosarcoma in osteosarcoma cell and organotypic cultures. However, studies in Col1/nHA 3D scaffold provided a contradictory result showing that the oxidative stress induced by PAR treatment favours tumour progression by enhancing cancer stem cell phenotype (70, 71). Incorporating nHA to PLGA fibres decreased the fibre diameter, produced a rough surface and improved the mechanical properties (tensile strength and modulus). Breast cancer cell line MCF7 grown on nHA/PLGA scaffold exhibited increased cell viability and growth, but control over DNA synthesis and cell division were observed compared to the cells cultured on PLGA scaffold. This discrepancy can be due to the direct correlation between breast cancer cells and the bone component, hydroxyapatite. The nHA/PLGA 3D scaffold model could be an excellent model to study breast cancer bone metastasis (72).

## Anti-Cancer Drug Validation Using *In Vitro* 3D Scaffold Models

Target identification, lead discovery, optimization, preclinical validation, and clinical trials are all steps of drug development that lead to approval for therapeutic use. The development of anti-cancer drugs with high efficacy and low toxicity is costly and highly challenging. Only one in ten drugs that move to clinical trials get approval from the FDA. The main reason for the high attrition rate is the lack of adequate preclinical models. Conventionally, 2D models do not mimic the tumor microenvironment; thus, most of the successful drugs in 2D conditions fail in clinical trials. It is essential to validate the anti-cancer drugs in a preclinical model that mimics the *in vivo* tumor microenvironment. Most of the anti-cancer drugs validated in the *in vitro* 3D scaffold model show an increased drug resistance compared with the 2D culture making them an ideal *in vitro* model for drug validation. The recent reports of *in vitro* 3D scaffold models in anti-cancer drug validation are listed in **Table 3**.

**Table 3:** Anti-cancer drugs screened using 3D tissue-engineered models in different cancer types.

Sl. No	Cancer type	Cell lines	Drugs	3D scaffold model	Effect	References
1	Lung cancer	A549	Doxorubicin, cisplatin, curcumin, paclitaxel, etoposide, 5-fluorouracil, and gemcitabine	Porous polymeric microspheres/microparticles	Increased drug resistance	(68, 69)
		A549	Cilengitide	Fibroblast layered polystyrene scaffold	• Inhibit cell adhesion • No cell cytotoxic effects	(73)
2	Breast cancer	MCF7	Doxorubicin, 5-fluorouracil, paclitaxel, (Z)-4-Hydroxytamoxifen (4OHT), fulvestrant, palbociclib	Patient-derived scaffold	• Higher doses of doxorubicin inhibit cell proliferation and CSC phenotype • 5-Fluorouracil decreased cell proliferation but a slight increase in CSC phenotype was observed • Paclitaxel has little or no effects on cell proliferation and CSC phenotype • Increased drug resistance to 4OHT and fulvestrant with an increase CSC phenotype • Palbociclib decreased cell proliferation	(57, 74)
		MCF7	5-Fluorouracil	Decellularized tissue matrix	• Increased CSC phenotype • Increased drug resistance with decreased apoptosis rate	(6, 75)
		MCF7	Doxorubicin	Fibrous scaffold	Increased drug resistance	(76)
		HCC1954	Doxorubicin and Bleomycin	Electrospun PCL based scaffold	• Cells were less sensitive to doxorubicin cytotoxic effects and showed an increased drug resistance. • Bleomycin alone does not affect cell viability • Cells were less susceptible to electroporation/bleomycin cytotoxic effects and showed an increased drug resistance	(62)
		EMT6	Doxorubicin	Silk fibroin scaffold	Cells were less sensitive to the cytotoxic effects of doxorubicin	(12)
		MCF7	Geniposide	3D printed hydrogel-based scaffold	Inhibit cell proliferation and induce apoptosis	(77)
		MCF7	Cilengitide	Fibroblast layered polystyrene scaffold	• Inhibit cell adhesion • No cell cytotoxic effects	(73)
		Primary breast cancer cells from patients	Doxorubicin and mitoxanthrone	Porous PCL based scaffold	Effect of drug varied with patient samples	(78)
3	Gastric cancer	AGS	Cisplatin	Porous silk scaffold	• Cisplatin loaded nanocomposite silk hydrogel showed an increase in shelf life • Gastric cancer cells were more sensitive to cisplatin nanocomposite silk hydrogel cytotoxic effects and prevented gastric cancer recurrence	(79)
4	Neuroblastoma	Kelly, KellyCis83	Cisplatin	Collagen-based scaffold	Increased drug resistance	(80)
5	Colorectal cancer	HT29, HCT116	5-Fluorouracil	Patient-derived scaffold	• Cells less sensitive to 5-fluorouracil cytotoxic effects • Increased drug resistance	(81, 82)
6	Ovarian cancer	OV-NC, OV-206	Carboplatin, 5-fluorouracil, and paclitaxel	Collagen-based scaffold	Increased drug resistance.	(28)
		HO-8910PM	Cisplatin, and paclitaxel	Hydrogel-based scaffold	Increased drug resistance	(27)
		R182	Docetaxel, cisplatin and doxorubicin	Collagen-based hydrogel scaffold	• Decreased apoptosis • Increased drug resistance	(83)
7	Bladder cancer	5637, T24	Rapamycin, BCG	3D printed hydrogel-based scaffold	Increased drug resistance and cells showed less sensitivity to both the drugs	(37)
8	Acute lymphoblastic leukaemia (ALL)	Jurkat	Cytarabine and daunorubicin	Collagen coated PCL scaffold	Increased drug resistance	(8)
9	Glioma	U118	Temozolomide	3D printed hydrogel-based scaffold	Increased drug resistance	(55)

## Scaffold-Based 3D Cellular Models for Primary Cancer Cell Culture

3D scaffolds can mimic the extracellular matrix, providing tumor cells structural support and particular physicochemical and biomechanical stimulation (84). A variety of 3D scaffold-based *in vitro* models has been processed to investigate the development of various cancer types and understand the influence of the cancer microenvironment on cellular responses when modelling cancer *in vitro.* Landberg et al. used primary breast tumors infiltrated with breast cancer cell lines to create cell-free patient-derived scaffolds (PDSs). Significant changes in differentiation, epithelial-mesenchymal transition, stemness and proliferation of the breast cancer cell population were determined. Interestingly, the global gene expression profile of PDS cultures was found to be similar to xenograft cultures confirming that the PDS model mimics *in vivo*-like growth conditions (85). Hume et al. developed a collagen-based scaffold that can recapitulate the stromal microenvironment by culturing breast tumor fragments and adipocytes in an anisotropic collagen scaffold. The cultured tumor fragments exhibited a distinct migratory phenotype and varying responses to anti-metastatic drugs, GM6001 (MMP inhibitor), Y-27632 (Rho-associated protein kinase inhibitor) and Canertinib. This model replicates the patient tumor as well as the TME. Culturing patient-derived tumor biopsy fragments in this scaffold can be a potential approach for developing breast cancer personalized therapies (9). Smiths et al. constructed implantable biopolymer devices that deliver CAR T cells directly to the surfaces of solid tumors, thereby exposing them to high concentrations of immune cells for a substantial period (86). Nayak et al. developed porous PCL scaffolds for culturing primary breast cancer cells and a CAF matrix layer. The CAF combined with the mechanical properties of the scaffolds presented a unique environment to the primary cancer cells, which led to enhanced cellular viability, attachment, and tumoroid formation. The primary cells showed higher viability on the hybrid scaffolds with enhanced cell-matrix interactions than bare scaffolds. Primary breast cancer cells in PCL exhibited slow degradation kinetics and in this study, we observed that the mechanical integrity of the scaffolds was preserved throughout processes of the growth of CAFs, decellularization and the growth of primary breast cancer cells. Drug response assays indicated that the patient-derived tumoroids on the hybrid scaffolds could capture the inter- and intra-patient heterogeneity properties (78). The patient-derived tumors on hybrid scaffolds could serve as an ideal platform for personalized medicine and could be employed to track the growth of the cells and response to chemotherapeutic agents and understand the mechanisms of drug resistance.

## Discussion

Over the last decade, a great deal of evidence has emerged demonstrating the importance of tumor-stroma interactions in cancer development, metastasis, and drug resistance. The long-term objective is to understand better how these interactions work to reverse the microenvironment’s tumor-advancing effects. For that reason, biologically appropriate 3D *in vitro* models are necessary. This area has been transformed by integrating technological advancements like polymeric scaffolds, 3D bioprinting platforms etc. Furthermore, the pharmaceutical sector is interested in using human patient-derived primary cells in these model. It will help investigate medications and personalized cancer therapies that can interfere in tumor–stroma interactions. A biocompatible and biodegradable 3D scaffold system incorporating the natural characteristics of tumor-specific ECM would maximize mimicry and the power of *in vitro* studies. Although 3D culture is typically superior to 2D culture, biological indicators from cells grown in 3D systems can be confusing depending on the scaffolding materials and model designs. This is understandable given that many different scaffolding materials, such as collagen, fibronectin etc., activate the functionally diverse receptors. Therefore, selecting appropriate 3D systems to address specific questions remains a challenge for the scaffold-engineering field. Another limitation in using a 3D scaffold system is understanding how patient heterogeneity, where tumors show a significant degree of heterogeneity in terms of mutations, tumor stroma composition etc., could be introduced effectively and whether the 3D scaffold system can successfully evaluate the efficacy of cancer immunotherapies. Despite these limitations, 3D models provide a more realistic starting point for understanding the cellular and molecular pathways involved in cancer cell/biomatrix interactions, especially in the CSC population and emulating the TME.

## Author Contributions

KU, LVT, and RMRK contributed to the first draft of the manuscript and literature review. All authors contributed to the article and approved the submitted version.

## Conflict of Interest

The authors declare that the research was conducted in the absence of any commercial or financial relationships that could be construed as a potential conflict of interest.

## Publisher’s Note

All claims expressed in this article are solely those of the authors and do not necessarily represent those of their affiliated organizations, or those of the publisher, the editors and the reviewers. Any product that may be evaluated in this article, or claim that may be made by its manufacturer, is not guaranteed or endorsed by the publisher.
